# The development of a set of key points to aid clinicians and researchers in designing and conducting n-of-1 trials

**DOI:** 10.1186/s13063-024-08261-z

**Published:** 2024-07-11

**Authors:** Robin Chatters, Olivia Hawksworth, Steven Julious, Andrew Cook

**Affiliations:** 1 Sheffield Clinical Trials Research Unit (CTRU), Sheffield Centre for Health and Related Research (SCHARR), The University of Sheffield, Sheffield, UK; 2 Sheffield Centre for Health and Related Research (SCHARR), The University of Sheffield, Sheffield, UK; 3 Clinical Trials Unit, University of Southampton, Southampton, UK

**Keywords:** n-of-1, Personalised medicine, Key points, Cross-over trials, Rare diseases

## Abstract

**Introduction:**

n-of-1 trials are undertaken to optimise the evaluation of health technologies in individual patients. They involve a single patient receiving treatments, both interventional and control, consecutively over set periods of time, the order of which is decided at random. Although n-of-1 trials are undertaken in medical research it could be argued they have the utility to be undertaken more frequently. We undertook the National Institute for Health Research (NIHR) commissioned DIAMOND (Development of generalisable methodology for n-of-1 trials delivery for very low volume treatments) project to develop key points to assist clinicians and researchers in designing and conducting n-of-1 trials.

**Methods:**

The key points were developed by undertaking a stakeholder workshop, followed by a discussion within the study team and then a stakeholder dissemination and feedback event. The stakeholder workshop sought to gain the perspectives of a variety of stakeholders (including clinicians, researchers and patient representatives) on the design and use of n-of-1 trials. A discussion between the study team was held to reflect on the workshop and draft the key points. Lastly, the stakeholders from the workshop were invited to a dissemination and feedback session where the proposed key points were presented and their feedback gained.

**Results:**

A set of 22 key points were developed based on the insights from the workshop and subsequent discussions. They provide guidance on when an n-of-1 trial might be a viable or appropriate study design and discuss key decisions involved in the design of n-of-1 trials, including determining an appropriate number of treatment periods and cycles, the choice of comparator, recommended approaches to randomisation and blinding, the use of washout periods and approaches to analysis.

**Conclusions:**

The key points developed in the project will support clinical researchers to understand key considerations when designing n-of-1 trials. It is hoped they will support the wider implementation of the study design.

**Supplementary Information:**

The online version contains supplementary material available at 10.1186/s13063-024-08261-z.

## Introduction

N-of-1 trials are designed to optimise the evaluation of health technologies in individual patients [1]. n-of-1 trials were first described in the medical literature in 1986 by Guyatt and colleagues as double-blind, randomised trials in which a single patient undergoes a series of pairs of treatments — a drug and placebo [2]. Variations on this design have since been described and are apparent in the literature.

It has been argued that n-of-1 trials are an underused design for the evaluation of health technologies in individual patients [3]. A number of barriers to their implementation might be behind this underuse, including lack of time of clinicians and resources to undertake such trials [4, 5], the high cost of undertaking such trials compared to the standard of care [5], and the concerns of patients about the effect of the trial on their health [4, 6]. A major barrier is a lack of researcher expertise in their design [5, 7], which may prevent them from undertaking n-of-1 trials, or lead to poorly designed or conducted investigations.

The DIAMOND (development of a generalisable methodology for n-of-1 trials delivery for very low volume treatments) project was commissioned by the National Institute for Health Research (NIHR) clinical trials unit (CTU) Support Funding scheme to develop a set of key points to assist clinicians and researchers in designing and conducting n-of-1 trials.

## Methods

The key points were developed initially through a workshop with key stakeholders, followed by a review of the key points by the study team, culminating in a dissemination and feedback event where the key points were reviewed by a wider group of stakeholders. A full report of the project has been published elsewhere [8].

### Stakeholder workshop

Firstly, a stakeholder workshop was conducted on 21^st^ January 2022, in which key principles of the design and implementation of n-of-1 trials were discussed, in light of the findings from an extensive review of n-of-1 trials [9]. The workshop aimed to seek the perspectives of a range of stakeholders on key elements of the design and implementation of n-of-1 trials and to generate discussion about the types of questions n-of-1 trials can be used to address, the treatments they can be used to evaluate and their possible outcomes.

#### Attendee selection and recruitment

We sought a range of stakeholders to participate in the workshop, including clinicians who have used (or may wish to use) n-of-1 trials, academics (statisticians, methodologists, researchers) with an interest and/or experience in n-of-1 trials and patient representatives who have participated in an n-of-1 trial and/or have experience of a rare disease.

A convenience sampling frame was used to identify potential attendees. Individuals were identified from existing contacts known by the research team, in addition to identifying additional stakeholders through online advertisements (Twitter, NIHR People in Research (https://www.peopleinresearch.org)), checking publications relating to n-of-1 trials and their associated list of authors to identify those that have published work in this area, research networks, and by contacting those who may use the findings from n-of-1 trials (e.g. GPs).

Stakeholders were approached via email and were provided with a short description of the project and workshop. Prior to the workshop, participants were provided with an information sheet explaining the purpose and format of the session. The majority of stakeholders did not have a relationship with the study team prior to the workshop, except for two attendees who had collaborated with co-facilitator SAJ on an n-of-1 trial. OH met with the patient representatives to provide some background information about n-of-1 trials and answer any questions they had, in order that they felt able to engage in the workshop discussions. All stakeholders would have been aware of the facilitator’s reasons for undertaking the workshop.

#### Structure of the workshop

Due to COVID-19 restrictions, the workshop was held online via video conferencing and was recorded to ensure accurate note-taking. Verbal consent was obtained at the start of the workshop. The workshop consisted of three main sections — a presentation of the findings from the review [9], followed by a breakout session where the key points for n-of-1 trials were discussed and a group feedback session where the discussions within the breakout groups were shared and explored.

Individuals were pre-assigned to one of three breakout groups, consisting of two groups of academics/researchers and one group of patient representatives. Individuals in the academic/researcher groups were allocated across two groups to ensure a mix of experience and knowledge. Six members of the study team facilitated the groups — OH (a female research assistant with a BSc), SAJ (a male professor with a PhD), SS (a male professor with a PhD), AC (a male public health physician with a medical degree), and RC (a male research fellow with a BSc). All facilitators were interested in the research topic and may be considered biased in their opinion of the importance of n-of-1 trials as a method to evaluate health technologies. To guide the discussions each group worked through a set of questions (see Additional file 1), which were piloted within the study team prior to the workshop. Each set of questions differed slightly between the academic/researcher and patient representative groups. However, both sets of questions covered when it is appropriate to undertake n-of-1 trials, the questions that can be addressed, the treatments that can be assessed and the barriers and challenges of undertaking such trials. In the group feedback session that followed, each group fed back a summary of their discussions on each question, with discussion facilitated by members of the study team (RC and SAJ).

#### Participants

Twenty-nine stakeholders were invited to the workshop. Sixteen individuals either did not reply (*n* = 13) or replied to say they were unable to attend (*n* = 3). The workshop was attended by 13 stakeholders (in addition to the six facilitators). Of the 13 stakeholders, nine were clinicians/academics, two were statisticians and two were Patient and Public Involvement (PPI) representatives living with a rare disease (Table 1).

**Table 1:** Demographics of workshop attendees

Participant ID number	Role	Gender
P1	Clinician/academic	Male
P2	Clinician/academic	Female
P3	Statistician	Female
P4	Clinician/academic	Female
P5	Statistician	Male
P6	Clinician/academic	Female
P7	Clinician/academic	Female
P8	Clinician/academic	Male
P9	Academic	Female
P10	Academic	Female
P11	Clinician/academic	Male
P12	Patient representative	Female
P13	Patient representative	Female

#### Data collection and analysis

Video recordings were made of the workshop and breakout discussions. OH listened to the recordings and made detailed notes from each of the discussions, capturing differing perspectives, themes, and any examples used to illustrate a key point. Themes were identified from the data and not in advance. The notes were collated and grouped according to the main themes of discussion that emerged from the workshop: the questions that can be addressed, the treatments that can be investigated, the outcomes that can be assessed, benefits, or challenges.

#### Study team discussion

Based on the discussions from the stakeholder workshop a draft of the key points for n-of-1 study design and implementation were developed. A meeting was held between the study team (SAJ, RC, OH, AC) via Google Meet in which draft key points were discussed and developed. Feedback from SS was obtained via email.

### Dissemination and feedback event

The dissemination and feedback event was held on 27^th^ June 2022, with the aim to share and obtain feedback on the draft key points with the stakeholders who attended the workshop.

An event was held online and which draft key points were presented and feedback obtained. All those that were invited to attend the initial workshop (including those that could not attend) were invited. The event involved SAJ, RC and OH presenting the draft key points to attendees, with time allocated for feedback from the attendees, which was facilitated by SAJ and RC. The event was recorded to allow OH to take notes from the session and make the necessary changes to the key points.

#### Participants and data collection

Eight individuals were unable to attend the event. Five stakeholders attended, including a patient representative, a statistician and three researchers in rare diseases, two of whom had experience of undertaking an n-of-1 trial (see Table 2).

**Table 2:** Demographics of attendees at the dissemination and feedback event

Participant ID number	Role	Gender
P4	Clinician/academic	Female
P5	Statistician	Male
P7	Clinician/academic	Female
P10	Academic	Female
P12	Patient representative	Female

#### Dissemination reflection

Participants at the dissemination and feedback event gave advice on the key points with respect to wording and highlighted the importance of public and patient involvement—an important point which was missing in the initial draft points. Based on the feedback a final document of key points was drafted.

## The DIAMOND key points for n-of-1 trials

The discussions led to the development of 22 key points to consider when designing and conducting n-of-1 trials, which are detailed in the following section. The key points are subdivided into two sections and the sections have been subdivided further into 12 themes, as described in Table 3. A checklist, which can be used to consider whether a given n-of-1 trial adheres to the key points, can be found in Additional file 2.

**Table 3:** DIAMOND key points — sections and themes

Section	Theme
Section 1: When it is appropriate to under an n-of-1 trial?	Scope
	Prevalence of health condition
	Type and attributes of health technologies
	Questions that can be addressed
Section 2: Design and analysis considerations for n-of-1 trials	Choice of outcome
	Choice of comparator
	Target of treatment
	Number of health technologies and periods
	Blinding
	Randomisation
	Analysis
	Patient and public involvement (PPI)

The full list of key points is given in Table 4.

**Table 4:** DIAMOND key points

Section and theme	Key point Number	Key point
**When it is appropriate to under an n-of-1 trial?**		
Scope	1	n-of-1 trials should primarily be used to inform decisions about the care of an individual patient
Prevalence of health condition	2	n-of-1 trials can be a viable study design for very low-volume interventions, such as those in rare (and ultra-rare) diseases
Type and attributes of health technologies	3	A wide range of health technologies can be assessed using n-of-1 trials, provided they meet the criteria specified in *key points 4 and 5*
	4	Health technologies to be assessed using n-of-1 trials must have an onset of effect that can feasibly be observed in a study period
	5	Health technologies to be assessed using n-of-1 trials must not have prolonged carryover effects
	6	n-of-1 trials might be appropriate for the investigation of expensive health technologies or those with significant side effects which effect users to differing extents
Questions that can be addressed	7	n-of-1 trials are appropriate when aiming to address one of four questions (see elaboration for details)
**Design and analysis considerations for n-of-1 trials**		
Choice of outcome	8	The question being addressed will inform the choice of primary outcome
	9	It is recommended to use both patient-reported outcome measures (PROMs) and more objective measures of effect where possible, especially in those trials that are being undertaken to assess the efficacy of an expensive or risky treatment
	10	n-of-1 trials can be used not only to assess the effect of a health technology on a primary efficacy outcome but also other outcomes which are important to the patient
Choice of comparator	11	The choice of comparator should be made to answer the research question for the study
Target of treatment	12	n-of-1 trials are used to provide evidence which can be used to improve the condition of the patient itself, specific symptoms of the condition, side effects, or patient satisfaction
Number of health technologies and periods	13	n-of-1 trials typically compare two health technologies. Designing n-of- 1 trials which compare three or more health technologies is associated with practical challenges
	14	The number of study periods in an n-of-1 trial is a trade-off between precision and feasibility
Blinding	15	n-of-1 trials should be blinded where feasible
Randomisation	16	Blocked randomisation of treatment allocation is typically recommended
Analysis	17	An interim analysis may be considered when designing n-of-1 trials. The analysis can be used to indicate whether early stopping of the trial is appropriate
	18	Washout periods or active (analytical) washout should be employed if there are likely to be carryover effects of the health technology under investigation
	19	Clinical, in addition to statistical, significance should be used to help judge the effect of treatment
	20	Within-patient analysis of an n-of-1 trial will determine whether a clinically important effect has been observed
	21	Between-patient analysis of a series of n-of-1 trials can be used to estimate the average treatment effect across all the trials; determine whether these effects are consistent for all of the patients and to estimate the average treatment effect for that population/sub-population generally
Patient and public input (PPI)	22	Relevant and meaningful PPI should be sought throughout the n-of-1 trial including design and planning; interpretation; dissemination and implementation

### DIAMOND key points explanation and elaboration

Where applicable, case studies are provided to provide exemplars for specific key points. A list of case studies, and the key points they relate to, can be found in Table 5. It should be noted that studies signified as not meeting certain key points may be due to a lack of information in the available report(s).

**Table 5:** Characteristics of the case studies in relation to the DIAMOND key points for n-of-1 trials

		**Key point**																					
**Case study**		1	2	3	4	5	6	7	8	9	10	11	12	13^a^	14	15	16	17	18	19	20	21	22
1	Benhamou et al. [10]	X	X	X	X	X		X	X	X	X	X	X	2	X						X	X	
2	Roustit et al. [11]	X		X	X	X		X	X	X	X	X	X	3	X	X					X		
3	Frost et al. [12]	X		X	X	X		X	X		X	X	X	2	X	X			X		X		
4	Tian et al. [13]	X		X	X	X		X	X		X	X	X	2	X		X		X		X	X	
5	Rvachew et al. [14]	X		X	X	X		X	X	X	X	X	X	3	X		X				X		
6	ISRCTN17945917 [15]	X	X	X	X	X	X	X	X	X	X	X	X	2	X						X		
7	McGarry et al. [16]	X		X	X	X	X	X	X		X	X	X	2	X	X			X		X		
8	Tsiormpatzis et al. [17]	X		X	X	X		X	X	X	X	X	X	2	X						X		
9	Samuel et al. [18]	X		X	X	X		X	X		X	X	X	4	X	X			X	X	X	X	
10	Stunnenberg et al. [19]	X	X	X	X	X		X	X	X	X	X	X	2	X	X	X	X	X		X	X	
11	Graham et al. [20]	X	X	X	X	X		X	X	X	X	X	X	2	X						X		X
12	Santos et al. [21]	X		X	X	X		X	X		X	X	X	2	X	X			X		X		
13	Ferreira et al. [22]	X		X	X	X		X	X	X	X	X	X	2	X	X			X	X	X		
14	Joy et al. [23]	X		X	X	X		X	X	X	X	X	X	2	X	X			X	X	X	X	
15	Brannon et al. [24]	X		X	X	X		X	X	X	X	X	X	4	X						X		
16	Lipka et al. [25]	X	X	X	X	X		X	X	X	X	X	X	2	X	X	X		X		X	X	
17	Cha et al. [26]	X	X	X	X	X		X	X	X	X	X	X	3	X	X					X	X	
18	Germini et al. [27]	X		X	X	X		X	X	X	X	X	X	2	X	X			X				

^a^Number of trial arms

#### Scope

##### Key point 1

An n-of-1 trial should be undertaken where there is a decision to be made regarding the treatment of an individual patient. In some circumstances, an n-of-1 trial could provide sufficient evidence of effect for a health technology to be commissioned for that patient.

n-of-1 trials have particular utility where there is large variation in treatment efficacy from patient to patient and so decisions for individual patients are needed.

n-of-1 trials should not be confused with crossover trials — although they do share many of the same design characteristics. Crossover trials are used when a group level estimate of effect is the primary objective (i.e., such trials look to answer the question ‘what is the average effect in between groups?’), whereas for n-of-1 trials the primary objective is to interpret the treatment effect for individual patients (i.e. ‘what is the effect in each patient?’).

#### Prevalence of health condition

##### Key point 2

There are limited options for the assessment of efficacy for very low-volume interventions, such as health technologies for ultra-rare diseases, as the size of the patient population may make it impractical or infeasible to recruit the number of patients required for a conventional parallel-group trial. In these cases, n-of-1 trials may be a useful alternative to a conventional parallel-group trial as a means of increasing precision when cases are rare. For an example of an n-of-1 trial undertaken in a rare disease, see *case study 1* [10].

#### Type and attributes of health technologies

##### Key point 3

n-of-1 trials can be designed to assess a wide range of health technologies such as drug treatments (see *case study 2* [11]), medical devices (see *case study 3* [12]), dietary (see *case study 4* [13]) and behavioural interventions (see *case study 5* [14]), provided they meet the criteria specified in key points 4 (onset of effect) and 5 (carryover effects).

##### Key point 4

In order to be suitable for study using an n-of-1 trial, a health technology must have an onset of effect that is quick enough that it can be measured within a study period.

The onset of effect will impact the length of the periods in an n-of-1 trial and thus the length of the study overall.

##### Key point 5

In an n-of-1 trial, it is important that any carryover effects from one period have expired before an assessment of effect for a subsequent period is conducted. This is to ensure that any effects observed in this assessment can be attributed to the treatment condition of that period. Washout techniques (see key point 19) can be employed to ensure that sufficient time has elapsed for carryover effects to expire.

##### Key point 6

n-of-1 trials can be used to provide evidence of whether the benefits of a health technology outweigh its drawbacks for a particular patient.

For an expensive health technology, an n-of-1 trial might be used to assess whether a particular health technology is effective in a particular patient (see *case study 6* [15]). If the health technology produces a clinically meaningful effect in a patient, the cost of commissioning it for this patient might be justified. If the health technology does not produce a clinically meaningful effect in a patient, then the cost of the n-of-1 might be justified by preventing unnecessary costs of commissioning a treatment that does not result in a clinically meaningful improvement for a patient.

If a health technology has significant associated side effects which affect users to differing extents, an n-of-1 trial might be used to inform an understanding of the trade-off between benefits and harms for that individual patient. n-of-1 trials are unlikely to be implemented for common, safe, low-cost treatments.

#### Questions that can be addressed in an n-of-1 trial

##### Key point 7

The four questions that can be assessed within n-of-1 trials are:

1. *Does the health technology work at all?* An n-of-1 trial answering this question will likely be assessing a novel health technology for which there is no evidence in the patient population nor an existing treatment to which it could be compared (see *case study 7* [16]). If the n-of-1 trial was evaluating a drug treatment, the assessment may be of the investigative therapy against placebo (see *case study 8* [17]).
2. *Does the health technology work better than the existing treatment(s)?* It might be important to answer this question when there is an existing treatment option for a patient as well as a novel one to be assessed (see *case study 9* [18]). If the n-of-1 trial was evaluating a drug treatment, the assessment may be of the investigative therapy against an active treatment control.
3. *Which health technology is best for a particular patient?* This question may be asked in two situations:
  - Where a treatment has high patient-to-patient variability in efficacy — if there is more than one treatment option available with no clear rationale for which will be optimal for a particular patient, for example, because there is high interindividual variability in treatment response, an n-of-1 trial could be used to determine the treatment choice for each patient (see *case study 10* [19]).
  - Where a number of treatment options are equally efficacious — here a decision will need to be made about on outcomes other than the primary efficacy outcome including factors like patient preference.
4. *Does the efficacy of the treatment vary between individuals?* A series of n-of-1 trials would be required to answer this type of question, where each of the individual trials would be answering one of the other questions above. For example, an n-of-1 study could establish the treatment effect of a novel drug treatment compared to placebo for an individual patient. If this were conducted in a number of patients, an assessment could be made of whether the effect is consistent across all patients or within a particular subgroup of patients.

#### Choice of outcome

##### Key point 8

For most questions, an efficacy outcome will be used as the primary outcome (see Table 6). The choice of efficacy outcome may be influenced by practical considerations such as the time to onset of effect. If the time to onset of effect is long, then an outcome that is usually a secondary outcome (i.e. a surrogate) could be the primary outcome for the study. For example, an early time point assessment of the primary outcome could be used if this is predictive of the final response.

**Table 6:** Design considerations for n-of-1 trials

Question^a^	Does the health technology work at all for a particular patient?	Does the health technology work better than the existing treatment(s) for a particular patient?	Which health technology is best for a particular patient?		Does the individual treatment effect vary between patients?
			**When there is high variability in effect between patients**	**When there is a number of equally efficacious treatments**	
**Design**	Individual n-of-1 trial	Individual n-of-1 trial	Individual n-of-1 trial	Individual n-of-1 trial	Series of n-of-1 trials
**Primary outcome**^b^	Efficacy	Efficacy	Efficacy	Patient preference	Efficacy
**Control**^c^	Placebo (drug trial)/standard of care (behavioural or other trial)	Active treatment	Active treatment	Active treatment	Placebo/standard of care/active treatment

^a^See key point 7

^b^see key points 8, 9 and 10

^c^see key point 11

Clinical biomarkers could also be used as the efficacy outcome if these are predictive of the efficacy effect. If an assessment of efficacy is not the primary research question, then patient preference (see *case study 11* [20]) or quality of life could be the primary outcome. Efficacy outcomes could be secondary outcomes in such a study.

##### Key point 9

Those trials that are being undertaken to assess the efficacy of an expensive or risky treatment may require more stringent design considerations than those trials that are being undertaken on a more informal basis to dictate care (see also *blinding – key point 20*). An example of this is *case study 6* [15].

In such a situation, sufficient evidence of clinical improvement is required. For example, just collecting patient preference may not be sufficient, but more objective measures of a clinically significant effect may be required, as well as PROMs.

##### Key point 10

n-of-1 trials can be used not only to assess the effect of a health technology on the primary efficacy outcome, but also other outcomes which are important to the patient. Such outcomes could be the primary outcome, or secondary outcomes, for the trial. For an example, see *case study 11* [20]. For example, an n-of-1 trial could be used to assess the effect of different therapies on treatment side effects. Alternatively, patient preference for care delivery might be assessed. n-of-1 trials might make the personalisation of outcomes possible.

#### Choice of comparator

##### Key point 11

Different comparators are appropriate to answer different research questions. See elaboration of key point 7 and Table 4*.*

#### Target of treatment

##### Key point 12

An n-of-1 study can be undertaken to assess a health technology in the improvement of the:

- Condition/disease itself — the patient may benefit as their health condition may improve (see *case study 12* [21]);
- Symptoms of the condition — the patient may benefit as their quality of life may improve (see *case study 13* [22]);
- Side effects — the patient may benefit as their quality of life may improve but also their health condition may improve as the treatment may be better tolerated, improving adherence (see case study 14 [23]); and
- Patient satisfaction — if two health technologies have equal efficacy but with different posology patient preference could determine the choice of treatment. The patient may benefit as they get the treatment which works best for them in terms of their daily life.

#### Number of health technologies and periods

##### Key point 13

Most n-of-1 trials compare two health technologies (e.g. drug and placebo or two active treatments — see *case study 3* [12]). Designing these trials is more straightforward than those evaluating more than two health technologies, which incur greater practical and logistical challenges such as an increased study duration (see *case study 9* [18]).

It is possible to conduct n-of-1 trials evaluating more than three health technologies, particularly if the period and washout lengths are short (*see case study 15* [24]); however, it might be preferable to instead conduct more than n-of-1 trial.

##### Key point 14

The more study periods there are in an n-of-1 trial, the greater the precision in the evaluation of effect as there are more evaluations of the health technologies. However, decisions about the number of study periods must take into consideration the overall study length.

For some n-of-1 trials, having many study periods may be practicable. For others, it both might not be practical or even required — for a patient preference study, it might be possible to get an answer in just two study periods.

The DIAMOND review of n-of-1 studies found that the median number of study periods in an n-of-1 trial was six (see also *case study 13* [25]). This seems to represent a balance between precision and feasibility. The number of periods though will also be influenced by practical considerations — for example, if each period has a long duration then the overall study duration will need to be considered when determining the number of periods.

#### Blinding

##### Key point 15

Where feasible, n-of-1 trials should be double-blinded — for an example, see *case study 17* [26]. Blinding is more difficult in n-of-1 trials of certain types of health technologies, such as behavioural or dietary interventions.

Blinding may be challenging in n-of-1 trials of drug treatments due to difficulty obtaining a suitable placebo or due to obvious differences in the appearance or effects of active treatments to be compared.

Blinding may be more important in n-of-1 trials because of the crossover design. If a double-blind is not possible, n-of-1 trials may be conducted as single-blind or open-label trials. Even for open-label trials, it is optimal to incorporate some blinding, such as blind assessment of outcomes.

#### Randomisation

##### Key point 16

Randomising the sequence of treatment allocation has the advantage of evenly distributing (on average) both known and unknown confounding factors between the health technologies.

Blocking randomisation using a block size equal to the number of health technologies in the study has the advantage of preventing the generation of undesirable sequences such as AAAABBBB which would make the study sensitive to drop out as, if a patient dropped out halfway through the study, they would only have data from treatment condition A that could be analysed. A block size of two will produce a sequence such as ABBAABAB. In this example, if a patient dropped out of the study after two periods, it would still be possible to assess their experience of both health technologies.

Even if there is no patient dropout there could be issues with an allocation AAAABBBBB if there is a time effect for the underlying condition in the patient. A sequence of the form ABBAABAB would help to mitigate against this. A pitfall of randomising with a small block size is that the patient and clinicians are more likely to work out or guess the treatment allocation.

It is typically not possible to conceal the block size from a patient in an n-of-1 trial, in order to uphold the ethical and legal requirement of informed consent. The risk of working out or guessing the treatment allocation should be weighed against the risk of patient withdrawal.

#### Analysis

##### Key point 17

An interim analysis may be considered if there are six or more study periods and the study is long enough to assess the effect in the reduced number of periods. Continuing switching between treatments may become difficult if the patient is experiencing a noticeable improvement or deterioration in their symptoms under one particular treatment such that there is sufficient evidence of effect for an individual patient prior to the planned completion of the trial. The rationale for stopping the study early should be considered on a study-by-study basis and where possible should be pre-specified [28]. See key point 22, as information from other patients may inform the decision. See *case study 10* for an example study with interim analyses [19].

##### Key point 18

Where there are likely to be carryover effects from one study period to the next, a washout period should be implemented between study periods — for example, see *case study 18* [27]. The required length of washout will be determined by the characteristics of the intervention. Where it would be inappropriate to withdraw treatment for a period of washout, the use of active washout should be considered. This is where patients are switched immediately from one treatment to another (if safe to do so) but measurement starts once the effect of the previous treatment has disappeared and a steady state has been reached.

Although longer washout periods are generally desirable, it can potentially lead to harm for the patients if treatment was withdrawn and therefore full washout can raise ethical concerns.

An active washout therefore is a valid design when a full washout will lead to harm. With the design, the assumption is when we make the clinical assessments the efficacy will be for the treatment in that pathway.

It is worth noting that carry-over is not just influenced by treatment. For outcomes such as patient-reported outcomes, there can be psychometric carry-over as patients can recall how they responded in previous periods.

##### Key point 19

Determining the effect of treatment based solely on statistical significance should be avoided. Clinical significance should also be considered (see *case study 14* [23]). Where possible, a definition of a clinically important effect should be defined in the protocol, in order to introduce a degree of objectivity to an otherwise subjective assessment.

##### Key point 20

Within-patient analysis of an n-of-1 trial will determine whether a clinically important effect has been observed. Replication is informative in the assessment of response as it enables an assessment of the personalised response to treatment for an individual patient including if the effect is consistent or varies.

##### Key point 21

If a series of n-of-1 trials is conducted in which there is a consistent effect of treatment observed across all patients (or in a subgroup of patients), then it would be possible to combine the individual estimates of effect using meta-analysis to obtain an overall estimate of effect. See *case study 9* [18].

Quantifying the effect within individual patients is still the primary analysis (see key point 2), but a meta-analysis is informative.

These estimates will inform clinical practice overall — for example, a recommendation could be made for all patients to receive the new treatment including those who have been in an n-of-1, as if the effects are consistent outcomes seen overall can be used for individual patients.

#### Patient and public involvement (PPI)

##### Key point 22

PPI is especially important in n-of-1 trials due to their personalised nature. Input may be sought from the patient who will be taking part, disease-specific charities, affiliated support groups or hospital/Trust-specific advisory groups. During the design and planning of the trial, input may be sought into the patient-facing materials (e.g. PIS), outcomes and the treatment and follow-up regimes. During interpretation and dissemination, input may be sought into how the results are presented and shared with other patients.

## Discussion

In this manuscript, we present the findings of the DIAMOND project, in which we developed 22 key points for researchers and clinicians to consider when designing and conducting n-of-1 trials. The key points provide guidance as to when to use this n-of-1 trial methodology, how to design such trials and considerations for data analysis.

A strength of this project is that the key points were informed by a workshop that was attended by 13 external stakeholders (plus four members of the study team) from a range of backgrounds and disciplines, as well as two patient representatives. To ensure they were generalisable, the final list of key points for n-of-1 trials was also reviewed by a subset of the stakeholders at a dissemination and feedback event. Limitations include that, with only 13 external stakeholders, the results may not be generalisable compared to if a larger group of individuals were assembled. Due to the COVID-19 pandemic, we were unable to meet with stakeholders in-person and had to rely on online meetings. However, we ensured the meetings were as interactive as possible the interactions were likely to be different to those if the meetings had been held in person.

The DIAMOND study builds on other similar studies in the area of n-of-1 trials. A detailed report by Kravitz et al*.* (2014) also summarises key considerations for designing and conducting n-of-1 trials [29]. The Kravitz et al*.* report was commissioned by the US government and is therefore US-focused and aimed at a wide audience (including patients, statisticians, and researchers). In comparison, the DIAMOND study engaged with predominantly UK-based stakeholders to develop UK-focused key points for n-of-1 trials targeted specifically at clinicians or trial methodologists who are interested in undertaking such trials. DIAMOND builds on the work undertaken by Kravitz et al*.* by presenting additional key points, including a list of questions that can be addressed by n-of-1 trials (key point 8), the consideration of an interim analysis part way through an n-of-1 trial (key point 17) and how the cost of a new treatment may influence the design of the trial (key point 6).

Barriers remain that may hinder clinicians and researchers in undertaking n-of-1 trials. One such obstacle is likely to be seeking regulatory (e.g. ethical) approvals for n-of-1 trials, which has been reported by several authors [30–32]. There has been discussion in the literature regarding whether n-of-1 trials require review by an ethics committee, stemming from a debate regarding whether such studies are medical research or an optimised form of clinical care [31, 32]. Several papers, based on the regulatory contexts in the USA and Netherlands, have looked to clarify when ethical approvals are required for n-of-1 trials, with the inclusion of only one participant (i.e. a single n-of-1 trial) being an important exclusionary factor to requiring approvals [30, 31]. In the UK, according to the Medicine and Healthcare products Regulatory Agency (MHRA) algorithm, a study that involves the allocation of treatments decided in advance by a trial protocol requires MHRA regulatory approvals [33]. There are reporting standards for both the study protocol and analysis paper for n-of-1 studies [28, 34].

There are some methodological considerations discussed in the literature that are not covered by the DIAMOND key points, such as non-randomised sequence allocations. Randomising the allocation of treatment to period is an important technique to reduce bias in an n-of-1 trial, so much so that some consider randomisation a defining feature of an n-of-1 trial [2, 35]. Some authors, however, suggest that counterbalancing (generation of a balanced, non-randomised sequence of treatment periods, e.g. AB BA BA AB) is an appropriate alternative to randomisation when there are known time trends of the condition being studied (i.e. it is deteriorating) [29]. Whilst we do not suggest counterbalancing as an alternative approach, our key points recommend blocking the randomisation by the number of health technologies under investigation. This goes some way to balancing the sequence to control for a known effect of time on the condition of the patient, because a sequence such as AAABBB cannot be produced. Additionally, if time trends are a key concern, then a crossover design will not be an optimal method of evaluation of a treatment as they rely on the condition being stable throughout the evaluation.

Further research could involve using the DIAMOND key points to develop a tool that allows the construction of a tailored protocol for a particular type of n-of-1 study, therefore allowing researchers and clinicians to implement the design and DIAMOND key points for n-of-1 trials.

## Supplementary Information

Supplementary Material 1.

Supplementary Material 2.

Supplementary Material 3.

## Data Availability

The data used to develop the key points can be found in the full report [8].

## Abbreviations

DIAMOND: Development of generalisable methodology for n-of-1 trials delivery for very low volume treatments
GPs: General practitioner
MHRA: Medicine and Healthcare products Regulatory Agency
PIS: Participant information sheet
PPI: Patient and public involvement
PROMs: Patient-reported outcome measures
UK: United Kingdom
US: United States
