# Correction: The development of a set of key points to aid clinicians and researchers in designing and conducting n-of-1 trials

**DOI:** 10.1186/s13063-024-08453-7

**Published:** 2024-09-09

**Authors:** Robin Chatters, Olivia Hawksworth, Steven Julious, Andrew Cook

**Affiliations:** 1 Shefeld Clinical Trials Research Unit (CTRU), Shefeld Centre for Health and Related Research (SCHARR), The University of Shefeld, Shefeld, UK; 2 Shefeld Centre for Health and Related Research (SCHARR), The University of Shefeld, Shefeld, UK; 3 Clinical Trials Unit, University of Southampton, Southampton, UK

**Correction** **:** **Trials 25, 473 (2024)**

**https://doi.org/10.1186/s13063-024-08261-z**

Following the publication of the original article [1], we were notified that Additional file 2 had a few placeholders for the manuscript citation, which have now been added.

The original article has been corrected.
