# Supplementary material for: The development of a set of key points to aid clinicians and researchers in designing and conducting n-of-1 trials
Source: Trials. 2024 Jul 11;25:473. doi: 10.1186/s13063-024-08261-z (PMC11241860; doi:10.1186/s13063-024-08261-z)
Supplement: Supplementary file 1 — Supplementary Material 1. [file 13063_2024_8261_MOESM1_ESM.docx]

# Additional file

**Breakout group questions for academics/researchers:**

1. What is your experience of undertaking/participating in n-of-1 studies?
2. When is it appropriate to use n-of-1 studies? When is it inappropriate?
3. What treatments can be tested within n-of-1 studies? What treatments can’t be tested?
4. What questions can be addressed using n-of-1 studies? What questions can’t be answered?
5. What are the challenges/barriers to undertaking n-of-1 studies? How may we mitigate these challenges?
6. What are the benefits/pitfalls to participating in n-of-1 studies?
7. How should n-of-1 studies be designed?
8. What outcomes can be used in n-of-1 studies?
9. Who may fund n-of-1 studies?
10. What guidance/recommendations can we make regarding the above?
11. Are there any specific recommendations relating to rare diseases?

**Breakout room questions for patient representatives:**

1. When is it appropriate to use n-of-1 studies? When is it inappropriate?
2. What questions would you like to see addressed within n-of-1 studies? (curative, symptomology, QoL).
3. What types of health technologies should be tested in n-of-1 studies that would promote participation from patients?
4. What are the challenges/barriers to a patient in being in n-of-1 studies? How may we mitigate these challenges?
5. What are the considerations to designing n-of-1 studies? Study duration?  Clinical outcomes? Number of periods?
6. What are the benefits to participating in n-of-1 studies?

What guidance/recommendations can we make regarding the above?
