# Supplementary material for: The development of a set of key points to aid clinicians and researchers in designing and conducting n-of-1 trials
Source: Trials. 2024 Jul 11;25:473. doi: 10.1186/s13063-024-08261-z (PMC11241860; doi:10.1186/s13063-024-08261-z)
Supplement: Supplementary file 2 — Supplementary Material 2. [file 13063_2024_8261_MOESM2_ESM.docx]

# Key points for n-of-1 trials

For a further elaboration of each key point please refer to: *Chatters, R., Hawksworth, O., Julious, S. et al. The development of a set of key points to aid clinicians and researchers in designing and conducting n-of-1 trials. Trials****25****, 473 (2024). https://doi.org/10.1186/s13063-024-08261-z*

| **Points to Consider** | | | **Checkbox** | |
| --- | --- | --- | --- | --- |
| **Section 1: When is it appropriate to undertake n-of-1 trials?** | | | | |
| **Scope** | | | | |
| 1 | n-of-1 trials should primarily be used to inform decisions about the care of an individual patient. | |  | |
| **Prevalence of health condition** | | | | |
| 2 | n-of-1 trials can be a viable study design for very low volume interventions, such as those in rare (and ultra-rare) diseases. | |  | |
| **Type and attributes of health technologies** | | | | |
| 3 | A wide range of health technologies can be assessed using n-of-1 trials, provided they meet the criteria specified in *points 4 and 5*. | |  | |
| 4 | Health technologies to be assessed using n-of-1 trials must have an onset of effect that can feasibly be observed in a study period. | |  | |
| 5 | Health technologies to be assessed using n-of-1 trials must not have prolonged carryover effects. | |  | |
| 6 | n-of-1 trials might be appropriate for the investigation of expensive health technologies or those with significant side effects which effect users to differing extents. | |  | |
| **Questions that can be addressed** | | | | |
| 7 | n-of-1 trials are appropriate when aiming to address one of four questions: 1) Does the health technology work at all? 2) Does the health technology work better than the existing treatment(s)? 3) Which health technology is best for a particular patient? 4) Does the efficacy of the treatment vary between individuals? For more information see *Table 4 [1].* | |  | |
| **Section 2: Design and analysis considerations for n-of-1 trials** | | | | |
| **Choice of outcome** | | | | |
| 8 | The question being addressed will inform the choice of primary outcome. For more information see *Table 4 [1]* | |  | |
| 9 | It is recommended to use both patient reported outcome measures (PROMs) and more objective measures of effect where possible, especially in those trials that are being undertaken to assess the efficacy of an expensive or risky treatment. | |  | |
| 10 | n-of-1 trials can be used not only to assess the effect of a health technology on a primary efficacy outcome but also other outcomes which are important to the patient. | |  | |
| **Choice of comparator** | | | | |
| 11 | The choice of comparator should be made to answer the research question for the study. | |  | |
| **Target of treatment** | | | | |
| 12 | | n-of-1 trials are used to provide evidence which can be used to improve the patient’s condition itself, specific symptoms of the condition, side effects, or patient satisfaction. | |  |
| **Number of health technologies and periods** | | | | |
| 13 | | n-of-1 trials typically compare two health technologies. Designing n-of-1 trials which compare three or more health technologies is associated with practical challenges. | |  |
| 14 | | The number of study periods in an n-of-1 trial is a trade-off between precision and feasibility. | |  |
| **Blinding** | | | | |
| 15 | | n-of-1 trials should be blinded where feasible. | |  |
| **Randomisation** | | | | |
| 16 | | Blocked randomisation of treatment allocation is typically recommended. | |  |
| **Analysis** | | | | |
| 18 | | An interim analysis may be considered when designing n-of-1 trials. The analysis can be used to indicate whether early stopping of the trial is appropriate. | |  |
| 19 | | Washout periods or active (analytical) washout should be employed if there are likely to be carryover effects of the health technology under investigation. | |  |
| 20 | | Clinical, in addition to statistical, significance should be used to help judge the effect of treatment. | |  |
| 21 | | Within-patient analysis of an n-of-1 trial will determine whether a clinically important effect has been observed. | |  |
| 22 | | Between-patient analysis of a series of n-of-1 trials can be used to estimate the average treatment effect across all the trials; determine whether these effects are consistent for all of the patients and to estimate the average treatment effect for that population/sub-population generally. | |  |
| **PPI** | | | | |
| 23 | | Relevant and meaningful PPI should be sought throughout the n-of-1 trial including design and planning; interpretation; dissemination and implementation. | |  |

**References**

[1] *Chatters, R., Hawksworth, O., Julious, S. et al. The development of a set of key points to aid clinicians and researchers in designing and conducting n-of-1 trials. Trials****25****, 473 (2024). https://doi.org/10.1186/s13063-024-08261-z*
